# Hexaaqua­magnesium bis­(3-carb­oxy-4-hy­droxy­benzene­sulfonate) dihydrate

**DOI:** 10.1107/S1600536811030777

**Published:** 2011-08-06

**Authors:** Graham Smith, Urs D. Wermuth, Michael L. Williams

**Affiliations:** a Faculty of Science and Technology, Queensland University of Technology, GPO Box 2434, Brisbane, Queensland 4001, Australia; b School of Biomolecular and Physical Sciences, Griffith University, Nathan, Queensland 4111, Australia

## Abstract

In the crystal structure of the title compound, [Mg(H_2_O)_6_](C_7_H_5_O_6_S)_2_·2H_2_O, the octa­hedral complex cation lies on an inversion centre and is hydrogen bonded through the coordinated water molecules to the substituted benzene­sulfonate monoanions and the water mol­ecules of solvation. These inter­actions together with a carb­oxy­lic acid O—H⋯O(sulfonate) association give a three-dimensional structure.

## Related literature

For the structure of the isotypic Mn^II^, Cu^II^ and Co^II^ dihydrate complexes, see: Ma *et al.* (2003*a* ▶,*d* ▶); Abdelhak *et al.* (2005 ▶). For the structures of the analogous Co^II^, Ni^II^ and Zn^II^ tetra­hydrate complexes, see: Ma *et al.* (2003*b* ▶,*c* ▶,*e* ▶).
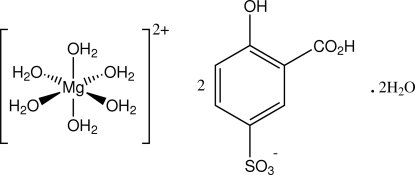


## Experimental

### 

#### Crystal data

[Mg(H_2_O)_6_](C_7_H_5_O_6_S)_2_·2H_2_O
*M* *_r_* = 602.78
Triclinic, 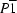

*a* = 6.8694 (4) Å
*b* = 6.9069 (4) Å
*c* = 14.3950 (8) Å
α = 77.472 (5)°
β = 78.120 (4)°
γ = 70.131 (5)°
*V* = 620.51 (6) Å^3^
*Z* = 1
Mo *K*α radiation
μ = 0.33 mm^−1^
*T* = 200 K
0.40 × 0.12 × 0.10 mm

#### Data collection

Oxford Diffraction Gemini-S CCD-detector diffractometer
Absorption correction: multi-scan (*CrysAlis PRO*; Oxford Diffraction, 2010 ▶) *T*_min_ = 0.96, *T*_max_ = 0.99
8134 measured reflections
2899 independent reflections
2553 reflections with *I* > 2σ(*I*)
*R*_int_ = 0.023

#### Refinement

*R*[*F* ^2^ > 2σ(*F* ^2^)] = 0.032
*wR*(*F* ^2^) = 0.086
*S* = 1.14
2899 reflections
209 parameters
H atoms treated by a mixture of independent and constrained refinement
Δρ_max_ = 0.34 e Å^−3^
Δρ_min_ = −0.43 e Å^−3^

### 

Data collection: *CrysAlis PRO* (Oxford Diffraction, 2010 ▶); cell refinement: *CrysAlis PRO*; data reduction: *CrysAlis PRO*; program(s) used to solve structure: *SIR92* (Altomare *et al.*, 1994 ▶); program(s) used to refine structure: *SHELXL97* (Sheldrick, 2008 ▶) within *WinGX* (Farrugia, 1999 ▶); molecular graphics: *PLATON* (Spek, 2009 ▶); software used to prepare material for publication: *PLATON*.

## Supplementary Material

Crystal structure: contains datablock(s) global, I. DOI: 10.1107/S1600536811030777/tk2770sup1.cif

Structure factors: contains datablock(s) I. DOI: 10.1107/S1600536811030777/tk2770Isup2.hkl

Additional supplementary materials:  crystallographic information; 3D view; checkCIF report

## Figures and Tables

**Table 1:** Hydrogen-bond geometry (Å, °)

*D*—H⋯*A*	*D*—H	H⋯*A*	*D*⋯*A*	*D*—H⋯*A*
O2—H2⋯O12	0.85 (2)	1.87 (2)	2.632 (2)	149 (2)
O11—H11⋯O53^i^	0.79 (3)	1.92 (3)	2.678 (2)	161 (3)
O1*W*—H11*W*⋯O12^ii^	0.85 (2)	1.93 (2)	2.779 (2)	175 (2)
O1*W*—H12*W*⋯O51	0.82 (3)	2.00 (3)	2.824 (2)	175 (2)
O2*W*—H21*W*⋯O4*W*^iii^	0.82 (3)	1.91 (3)	2.728 (3)	173 (3)
O3*W*—H31*W*⋯O51^iv^	0.75 (3)	2.10 (3)	2.850 (2)	171 (3)
O3*W*—H32*W*⋯O4*W*^v^	0.89 (3)	1.87 (3)	2.748 (3)	167 (2)
O4*W*—H41*W*⋯O53^vi^	0.79 (3)	2.04 (3)	2.803 (2)	162 (3)
O4*W*—H42*W*⋯O52	0.84 (3)	1.88 (3)	2.717 (2)	178 (3)

Symmetry codes: (i) 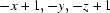; (ii) 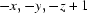; (iii) 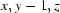; (iv) 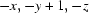; (v) 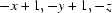; (vi) 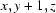.

**Fig. 1.**
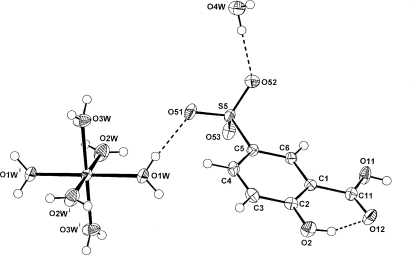
Molecular configuration and atom naming scheme for the cation, anion and water species in the asymmetric unit of the title compound. Inter-species hydrogen bonds are shown as dashed lines and displacement ellipsoids are drawn at the 50% probability level. For symmetry code (i): -x, -y, -z.

**Fig. 2.**
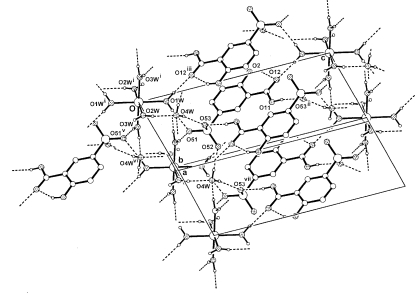
The hydrogen-bonding interactions in the title compound viewed down a. For symmetry codes, see Table 1.

## supplementary crystallographic information

### Comment

The title compound, [Mg(H_2_O)_6_]^2+^ 2(C_7_H_5_O_6_S^-^). 2(H_2_O) was obtained from the reaction of 3-carboxy-4-hydroxybenzenesulfonic acid (5-sulfosalicylic acid, 5-SSA) with MgCO_3_ and the structure is reported here In the structure of this compound (Fig. 1), the octahedral cationic Mg complex cations lie on crystallographic inversion centres [Mg—O, 2.0396 (17)–2.0664 (19) Å]. This complex is isomorphous with other divalent first transition metal–5-SSA complexes with the same basic dihydrate formula [*M*(H_2_O)_6_] 2(5-SSA^-^). 2(H_2_O), [*M* = Mn (Ma *et al.*, 2003*a*); Co (Abdelhak *et al.*, 2005); Cu (Ma *et al.*, 2003*d*)]. These complexes are also similar to the tetrahydrate analogues {[*M*(H_2_O)_6_] 2(5-SSA^-^). 4(H_2_O)}, having triclinic unit cells with comparable cell parameters *e.g.* Co^II^ (Ma *et al.*, 2003*b*) and Ni (Ma *et al.*, 2003*c*) and Zn (Ma *et al.*, 2003*e*).

The coordinated water molecules are involved in a number of O—H···O hydrogen-bonding interactions with sulfonate and carboxylate O acceptors of the uncoordinated 5-SSA monoanions and the water molecules of solvation (Table 1) and together with a carboxylic acid *O*—*H*···O_sulfonate_ hydrogen bond form a three-dimensional structure (Fig. 2). In the anion there is the intramolecular cyclic phenol *O*—*H*···O_carboxyl_ hydrogen bond which is invariably present in this monoanion (Ma *et al.*, 2003*a*). One H of one of the coordinated water molecules (H22W) has no reasonable acceptor in the structure.

### Experimental

The title compound was synthesized by heating 218 mg (1 mmol) of 3-carboxy-4-hydroxybenzenesulfonic acid (5-sulfosalicylic acid) with an excess of MgCO_3_ in 50 ml of 1:1 ethanol–water under reflux for 10 min. After completion of the reaction, the unreacted MgCO_3_ was removed by filtration and the solution was allowed evaporate to incipient dryness at room temperature, giving small colourless prisms of the title compound from which a specimen was cleaved for the X-ray analysis.

### Refinement

Hydrogen atoms involved in hydrogen-bonding interactions were located by difference methods and their positional and isotropic displacement parameters were refined. Other H-atoms were included in the refinement at calculated positions [C–H = 0.93 Å and with *U*_iso_(H) = 1.2*U*_eq_(C), using a riding-model approximation.

### Crystal data

**Table:**

[Mg(H_2_O)_6_](C_7_H_5_O_6_S)_2_·2H_2_O	*Z* = 1
*M**_r_* = 602.78	*F*(000) = 314
Triclinic, *P*1	*D*_x_ = 1.613 Mg m^−^^3^
Hall symbol: -P 1	Mo *K*α radiation, λ = 0.71073 Å
*a* = 6.8694 (4) Å	Cell parameters from 4714 reflections
*b* = 6.9069 (4) Å	θ = 3.2–28.9°
*c* = 14.3950 (8) Å	µ = 0.33 mm^−^^1^
α = 77.472 (5)°	*T* = 200 K
β = 78.120 (4)°	Prism, colourless
γ = 70.131 (5)°	0.40 × 0.12 × 0.10 mm
*V* = 620.51 (6) Å^3^	

### Data collection

**Table:**

Oxford Diffraction Gemini-S CCD-detector diffractometer	2899 independent reflections
Radiation source: Enhance (Mo) X-ray source	2553 reflections with *I* > 2σ(*I*)
graphite	*R*_int_ = 0.023
ω scans	θ_max_ = 28.8°, θ_min_ = 3.2°
Absorption correction: multi-scan (*CrysAlis PRO*; Oxford Diffraction, 2010)	*h* = −9→9
*T*_min_ = 0.96, *T*_max_ = 0.99	*k* = −9→8
8134 measured reflections	*l* = −19→18

### Refinement

**Table:**

Refinement on *F*^2^	Primary atom site location: structure-invariant direct methods
Least-squares matrix: full	Secondary atom site location: difference Fourier map
*R*[*F*^2^ > 2σ(*F*^2^)] = 0.032	Hydrogen site location: inferred from neighbouring sites
*wR*(*F*^2^) = 0.086	H atoms treated by a mixture of independent and constrained refinement
*S* = 1.14	*w* = 1/[σ^2^(*F*_o_^2^) + (0.0388*P*)^2^ + 0.10*P*] where *P* = (*F*_o_^2^ + 2*F*_c_^2^)/3
2899 reflections	(Δ/σ)_max_ = 0.001
209 parameters	Δρ_max_ = 0.34 e Å^−^^3^
0 restraints	Δρ_min_ = −0.43 e Å^−^^3^

### Special details

**Table:**

Geometry. Bond distances, angles *etc*. have been calculated using the rounded fractional coordinates. All su's are estimated from the variances of the (full) variance-covariance matrix. The cell e.s.d.'s are taken into account in the estimation of distances, angles and torsion angles
Refinement. Refinement of *F*^2^ against ALL reflections. The weighted *R*-factor *wR* and goodness of fit *S* are based on *F*^2^, conventional *R*-factors *R* are based on *F*, with *F* set to zero for negative *F*^2^. The threshold expression of *F*^2^ > σ(*F*^2^) is used only for calculating *R*-factors(gt) *etc*. and is not relevant to the choice of reflections for refinement. *R*-factors based on *F*^2^ are statistically about twice as large as those based on *F*, and *R*- factors based on ALL data will be even larger.

### Fractional atomic coordinates and isotropic or equivalent isotropic displacement parameters (Å^2^)

**Table:**

	*x*	*y*	*z*	*U*_iso_*/*U*_eq_	
S5	0.32291 (6)	0.27288 (6)	0.24107 (3)	0.0205 (1)	
O2	−0.30636 (17)	0.30257 (19)	0.58938 (9)	0.0293 (4)	
O11	0.33290 (17)	0.10533 (19)	0.60809 (9)	0.0282 (4)	
O12	0.00797 (17)	0.16915 (18)	0.68922 (8)	0.0285 (3)	
O51	0.21125 (18)	0.34405 (17)	0.15798 (8)	0.0282 (3)	
O52	0.4428 (2)	0.4036 (2)	0.24880 (9)	0.0358 (4)	
O53	0.45513 (17)	0.05481 (18)	0.24189 (8)	0.0288 (3)	
C1	0.0565 (2)	0.2280 (2)	0.51806 (10)	0.0168 (4)	
C2	−0.1584 (2)	0.2911 (2)	0.51141 (11)	0.0198 (4)	
C3	−0.2240 (2)	0.3455 (2)	0.42131 (12)	0.0235 (5)	
C4	−0.0799 (2)	0.3391 (2)	0.33881 (11)	0.0223 (4)	
C5	0.1338 (2)	0.2784 (2)	0.34503 (11)	0.0182 (4)	
C6	0.2005 (2)	0.2238 (2)	0.43408 (11)	0.0176 (4)	
C11	0.1284 (2)	0.1655 (2)	0.61309 (11)	0.0193 (4)	
Mg1	0.00000	0.00000	0.00000	0.0200 (2)	
O1W	0.0127 (2)	0.0693 (2)	0.12890 (9)	0.0323 (4)	
O2W	0.3193 (2)	−0.1459 (2)	−0.01676 (11)	0.0338 (4)	
O3W	0.0515 (2)	0.27534 (19)	−0.06749 (9)	0.0307 (4)	
O4W	0.5409 (2)	0.7353 (2)	0.13401 (10)	0.0313 (4)	
H2	−0.244 (3)	0.264 (3)	0.6383 (17)	0.045 (6)*	
H3	−0.36580	0.38640	0.41680	0.0280*	
H4	−0.12490	0.37510	0.27890	0.0270*	
H6	0.34260	0.18390	0.43800	0.0210*	
H11	0.369 (4)	0.070 (3)	0.6595 (18)	0.049 (7)*	
H11W	−0.002 (3)	−0.002 (3)	0.1845 (18)	0.049 (6)*	
H12W	0.067 (4)	0.155 (4)	0.1343 (17)	0.050 (7)*	
H21W	0.395 (4)	−0.180 (4)	0.025 (2)	0.061 (8)*	
H22W	0.383 (5)	−0.180 (4)	−0.068 (2)	0.083 (10)*	
H31W	−0.028 (4)	0.370 (4)	−0.0898 (17)	0.044 (7)*	
H32W	0.177 (4)	0.293 (4)	−0.0885 (17)	0.055 (7)*	
H41W	0.490 (4)	0.826 (4)	0.1655 (18)	0.058 (8)*	
H42W	0.514 (4)	0.631 (4)	0.1686 (18)	0.055 (7)*	

### Atomic displacement parameters (Å^2^)

**Table:**

	*U*^11^	*U*^22^	*U*^33^	*U*^12^	*U*^13^	*U*^23^
S5	0.0224 (2)	0.0251 (2)	0.0139 (2)	−0.0083 (2)	−0.0034 (1)	−0.0009 (1)
O2	0.0175 (6)	0.0402 (7)	0.0254 (7)	−0.0054 (5)	0.0016 (5)	−0.0055 (5)
O11	0.0199 (6)	0.0418 (7)	0.0185 (6)	−0.0022 (5)	−0.0075 (5)	−0.0035 (5)
O12	0.0263 (6)	0.0358 (6)	0.0176 (6)	−0.0060 (5)	−0.0002 (5)	−0.0007 (5)
O51	0.0354 (6)	0.0288 (6)	0.0177 (6)	−0.0063 (5)	−0.0106 (5)	0.0022 (4)
O52	0.0433 (7)	0.0494 (8)	0.0254 (6)	−0.0322 (6)	−0.0010 (6)	−0.0027 (5)
O53	0.0267 (6)	0.0321 (6)	0.0212 (6)	0.0025 (5)	−0.0061 (5)	−0.0074 (5)
C1	0.0171 (7)	0.0134 (6)	0.0189 (7)	−0.0034 (5)	−0.0042 (6)	−0.0014 (5)
C2	0.0177 (7)	0.0166 (7)	0.0237 (8)	−0.0040 (6)	−0.0007 (6)	−0.0048 (6)
C3	0.0154 (7)	0.0246 (8)	0.0301 (9)	−0.0028 (6)	−0.0067 (6)	−0.0058 (6)
C4	0.0218 (7)	0.0224 (7)	0.0223 (8)	−0.0029 (6)	−0.0105 (6)	−0.0025 (6)
C5	0.0192 (7)	0.0174 (7)	0.0173 (7)	−0.0052 (6)	−0.0030 (6)	−0.0017 (5)
C6	0.0152 (7)	0.0177 (7)	0.0192 (7)	−0.0038 (5)	−0.0044 (6)	−0.0016 (5)
C11	0.0206 (7)	0.0168 (7)	0.0190 (7)	−0.0033 (6)	−0.0036 (6)	−0.0029 (6)
Mg1	0.0227 (4)	0.0233 (4)	0.0142 (4)	−0.0084 (3)	−0.0045 (3)	0.0007 (3)
O1W	0.0492 (8)	0.0395 (7)	0.0156 (6)	−0.0245 (6)	−0.0067 (5)	−0.0005 (5)
O2W	0.0254 (6)	0.0455 (8)	0.0245 (7)	−0.0053 (6)	−0.0017 (6)	−0.0042 (6)
O3W	0.0247 (6)	0.0250 (6)	0.0371 (7)	−0.0078 (5)	−0.0063 (6)	0.0079 (5)
O4W	0.0300 (7)	0.0287 (7)	0.0357 (7)	−0.0114 (6)	−0.0048 (6)	−0.0022 (6)

### Geometric parameters (Å, °)

**Table:**

S5—O51	1.4584 (15)	O2W—H21W	0.82 (3)
S5—O52	1.4466 (17)	O2W—H22W	0.82 (3)
S5—O53	1.4699 (15)	O3W—H32W	0.89 (3)
S5—C5	1.7661 (19)	O3W—H31W	0.75 (3)
Mg1—O1W	2.0396 (17)	O4W—H42W	0.84 (3)
Mg1—O2W	2.0664 (19)	O4W—H41W	0.79 (3)
Mg1—O3W	2.0494 (17)	C1—C11	1.476 (2)
Mg1—O1W^i^	2.0396 (17)	C1—C6	1.394 (2)
Mg1—O2W^i^	2.0664 (19)	C1—C2	1.408 (2)
Mg1—O3W^i^	2.0494 (17)	C2—C3	1.393 (2)
O2—C2	1.347 (2)	C3—C4	1.379 (2)
O11—C11	1.314 (2)	C4—C5	1.399 (2)
O12—C11	1.229 (2)	C5—C6	1.382 (2)
O2—H2	0.85 (2)	C3—H3	0.9300
O11—H11	0.79 (3)	C4—H4	0.9300
O1W—H11W	0.85 (2)	C6—H6	0.9300
O1W—H12W	0.82 (3)		
			
S5···H12W	2.96 (3)	O1W···H22W^i^	2.83 (4)
S5···H42W	3.08 (3)	O2···H3^v^	2.5300
S5···H11^ii^	2.92 (2)	O2W···H32W	2.87 (3)
S5···H22W^iii^	2.91 (3)	O3W···H12W	2.86 (2)
O1W···O2W	2.907 (3)	O4W···H32W^x^	1.87 (3)
O1W···O3W	2.879 (2)	O4W···H21W^xi^	1.91 (3)
O1W···O51	2.824 (2)	O11···H6	2.3800
O1W···O12^iv^	2.779 (2)	O11···H6^ii^	2.5200
O1W···O2W^i^	2.900 (3)	O12···H11W^iv^	1.93 (2)
O1W···O3W^i^	2.903 (2)	O12···H2	1.87 (2)
O2···C3^v^	3.324 (3)	O51···H4	2.5600
O2···O11^vi^	3.151 (3)	O51···H31W^ix^	2.10 (3)
O2···O12	2.632 (2)	O51···H22W^iii^	2.78 (3)
O2···O52^vii^	3.207 (3)	O51···H12W	2.00 (3)
O2W···O3W^i^	2.926 (3)	O52···H6	2.8900
O2W···O1W	2.907 (3)	O52···H2^vii^	2.89 (2)
O2W···O3W	2.894 (2)	O52···H42W	1.88 (3)
O2W···O1W^i^	2.900 (3)	O53···H11^ii^	1.92 (3)
O2W···O4W^viii^	2.728 (3)	O53···H22W^iii^	2.61 (3)
O3W···O2W	2.894 (2)	O53···H41W^viii^	2.04 (3)
O3W···O1W	2.879 (2)	C1···C2^iv^	3.515 (3)
O3W···O51^ix^	2.850 (2)	C1···C1^vii^	3.511 (3)
O3W···O1W^i^	2.903 (2)	C1···C2^vii^	3.543 (3)
O3W···O2W^i^	2.926 (3)	C2···C1^vii^	3.543 (3)
O3W···O4W^x^	2.748 (3)	C2···C6^iv^	3.570 (3)
O4W···O2W^xi^	2.728 (3)	C2···C6^vii^	3.509 (3)
O4W···O53^xi^	2.803 (2)	C2···C1^iv^	3.515 (3)
O4W···O52	2.717 (2)	C3···C11^vii^	3.568 (3)
O4W···O3W^x^	2.748 (3)	C3···C11^iv^	3.490 (3)
O11···O2^xii^	3.151 (3)	C3···O2^v^	3.324 (3)
O11···O53^ii^	2.678 (2)	C4···C11^vii^	3.529 (3)
O11···C6^ii^	3.264 (3)	C4···C11^iv^	3.519 (3)
O12···O1W^iv^	2.779 (2)	C6···C2^iv^	3.570 (3)
O12···O2	2.632 (2)	C6···C2^vii^	3.509 (3)
O51···O1W	2.824 (2)	C6···O11^ii^	3.264 (3)
O51···O3W^ix^	2.850 (2)	C11···C4^vii^	3.529 (3)
O52···O4W	2.717 (2)	C11···C3^iv^	3.490 (3)
O52···O2^vii^	3.207 (3)	C11···C3^vii^	3.568 (3)
O53···O4W^viii^	2.803 (2)	C11···C4^iv^	3.519 (3)
O53···O11^ii^	2.678 (2)	C11···H2	2.38 (2)
O1W···H21W	2.92 (3)		
			
O51—S5—O52	114.10 (7)	Mg1—O2W—H21W	126.5 (19)
O51—S5—O53	110.20 (7)	H21W—O2W—H22W	113 (3)
O51—S5—C5	107.36 (7)	Mg1—O3W—H32W	125.3 (17)
O52—S5—O53	111.08 (8)	Mg1—O3W—H31W	126 (2)
O52—S5—C5	106.81 (7)	H31W—O3W—H32W	107 (3)
O53—S5—C5	106.91 (7)	H41W—O4W—H42W	105 (3)
O2W—Mg1—O3W^i^	90.64 (6)	C2—C1—C11	120.22 (13)
O1W^i^—Mg1—O3W	90.48 (5)	C6—C1—C11	120.43 (13)
O2W^i^—Mg1—O3W	90.64 (6)	C2—C1—C6	119.35 (13)
O3W—Mg1—O3W^i^	180.00	O2—C2—C1	122.58 (14)
O1W^i^—Mg1—O2W^i^	90.12 (6)	C1—C2—C3	119.67 (14)
O1W^i^—Mg1—O3W^i^	89.53 (5)	O2—C2—C3	117.75 (13)
O2W^i^—Mg1—O3W^i^	89.36 (6)	C2—C3—C4	120.41 (14)
O1W^i^—Mg1—O2W	89.88 (6)	C3—C4—C5	120.14 (14)
O1W—Mg1—O2W	90.12 (6)	C4—C5—C6	119.90 (14)
O1W—Mg1—O3W	89.53 (5)	S5—C5—C6	118.58 (11)
O1W—Mg1—O1W^i^	180.00	S5—C5—C4	121.52 (12)
O1W—Mg1—O2W^i^	89.88 (6)	C1—C6—C5	120.53 (14)
O1W—Mg1—O3W^i^	90.48 (5)	O11—C11—O12	123.56 (14)
O2W—Mg1—O3W	89.36 (6)	O11—C11—C1	113.42 (13)
O2W—Mg1—O2W^i^	180.00	O12—C11—C1	123.02 (14)
C2—O2—H2	107.2 (15)	C2—C3—H3	120.00
C11—O11—H11	112 (2)	C4—C3—H3	120.00
Mg1—O1W—H11W	128.1 (14)	C3—C4—H4	120.00
Mg1—O1W—H12W	123.6 (17)	C5—C4—H4	120.00
H11W—O1W—H12W	106 (2)	C1—C6—H6	120.00
Mg1—O2W—H22W	121 (2)	C5—C6—H6	120.00
			
O51—S5—C5—C4	1.22 (13)	C2—C1—C11—O11	178.30 (13)
O51—S5—C5—C6	−177.90 (11)	C2—C1—C11—O12	−1.5 (2)
O52—S5—C5—C4	124.00 (12)	C6—C1—C11—O11	−1.25 (19)
O52—S5—C5—C6	−55.12 (13)	C6—C1—C11—O12	178.95 (13)
O53—S5—C5—C4	−117.02 (12)	O2—C2—C3—C4	179.28 (13)
O53—S5—C5—C6	63.86 (13)	C1—C2—C3—C4	−0.4 (2)
C6—C1—C2—O2	−178.76 (13)	C2—C3—C4—C5	−0.2 (2)
C6—C1—C2—C3	0.9 (2)	C3—C4—C5—S5	−178.80 (11)
C11—C1—C2—O2	1.7 (2)	C3—C4—C5—C6	0.3 (2)
C11—C1—C2—C3	−178.65 (12)	S5—C5—C6—C1	179.34 (10)
C2—C1—C6—C5	−0.8 (2)	C4—C5—C6—C1	0.2 (2)
C11—C1—C6—C5	178.75 (12)		

Symmetry codes: (i) −*x*, −*y*, −*z*; (ii) −*x*+1, −*y*, −*z*+1; (iii) −*x*+1, −*y*, −*z*; (iv) −*x*, −*y*, −*z*+1; (v) −*x*−1, −*y*+1, −*z*+1; (vi) *x*−1, *y*, *z*; (vii) −*x*, −*y*+1, −*z*+1; (viii) *x*, *y*−1, *z*; (ix) −*x*, −*y*+1, −*z*; (x) −*x*+1, −*y*+1, −*z*; (xi) *x*, *y*+1, *z*; (xii) *x*+1, *y*, *z*.

### Hydrogen-bond geometry (Å, °)

**Table:**

*D*—H···*A*	*D*—H	H···*A*	*D*···*A*	*D*—H···*A*
O2—H2···O12	0.85 (2)	1.87 (2)	2.632 (2)	149 (2)
O11—H11···O53^ii^	0.79 (3)	1.92 (3)	2.678 (2)	161 (3)
O1W—H11W···O12^iv^	0.85 (2)	1.93 (2)	2.779 (2)	175 (2)
O1W—H12W···O51	0.82 (3)	2.00 (3)	2.824 (2)	175 (2)
O2W—H21W···O4W^viii^	0.82 (3)	1.91 (3)	2.728 (3)	173 (3)
O3W—H31W···O51^ix^	0.75 (3)	2.10 (3)	2.850 (2)	171 (3)
O3W—H32W···O4W^x^	0.89 (3)	1.87 (3)	2.748 (3)	167 (2)
O4W—H41W···O53^xi^	0.79 (3)	2.04 (3)	2.803 (2)	162 (3)
O4W—H42W···O52	0.84 (3)	1.88 (3)	2.717 (2)	178 (3)
C3—H3···O2^v^	0.93	2.53	3.324 (3)	144
C4—H4···O51	0.93	2.56	2.940 (3)	105
C6—H6···O11	0.93	2.38	2.705 (2)	100
C6—H6···O11^ii^	0.93	2.52	3.264 (3)	137

Symmetry codes: (ii) −*x*+1, −*y*, −*z*+1; (iv) −*x*, −*y*, −*z*+1; (viii) *x*, *y*−1, *z*; (ix) −*x*, −*y*+1, −*z*; (x) −*x*+1, −*y*+1, −*z*; (xi) *x*, *y*+1, *z*; (v) −*x*−1, −*y*+1, −*z*+1.
